# Delayed motherhood and maternal health risks in India: findings from a generalized linear model analysis

**DOI:** 10.3389/fpubh.2026.1769597

**Published:** 2026-03-20

**Authors:** Mayank Singh, Chander Shekhar, Jagriti Gupta

**Affiliations:** 1 KLE Academy of Higher Education and Research, Department of Epidemiology and Biostatistics, Belagavi, Karnataka, India; 2 Department of Fertility and Social Demography - International Institute for Population Sciences (IIPS), Mumbai, India; 3 Bagchi School of Public Health, Ahmedabad University, Gujarat, Ahmedabad, India

**Keywords:** delayed motherhood, generalized linear model, India, Maternal health outcomes, NFHS-5, Reproductive Health Services

## Abstract

**Introduction:**

Delayed motherhood, defined as childbirth at advanced maternal age (≥35 years), is a growing trend globally and increasingly observed in India, driven by shifts in socio-economic conditions, rising female education, and urbanization. While studies from high-income countries have linked advanced maternal age to adverse maternal outcomes, including nutritional risks and obstetric complications, evidence from nationally representative Indian data remains limited. Women aged 40–49 whose most recent birth likely represents their final reproductive event provide a unique opportunity to study delayed motherhood in the context of completed fertility.

**Data and methods:**

This study analyzed cross-sectional data from the National Family Health Survey-5 (NFHS-5, 2019–21). The analytical sample included 148,655 ever-married women aged 40–49 years. Delayed motherhood was defined as having the most recent childbirth at age ≥35, irrespective of parity. Maternal health outcomes included nutritional status (underweight, overweight), anemia prevalence, reproductive healthcare utilization (delivery care, sterilization), contraceptive use prior to first childbirth, unmet need for contraception, and pregnancy termination. Bivariate analyses were conducted initially, followed by generalized linear models (logistic regression) adjusting for demographic (age, residence, religion, caste) and socioeconomic (education, wealth index, employment) covariates.

**Results:**

Women experiencing delayed motherhood had significantly higher adjusted odds of being underweight (AOR: 1.15; 95% CI: 1.09–1.22) and having unmet contraceptive needs (AOR: 1.98; 95% CI: 1.89–2.09). They also showed increased odds of pregnancy termination (AOR: 1.43; 95% CI: 1.36–1.50) and using contraception prior to first childbirth (AOR: 1.23; 95% CI: 1.15–1.32). Conversely, delayed mothers were less likely to be overweight (AOR: 0.90; 95% CI: 0.86–0.94) or to have undergone sterilization (AOR: 0.35; 95% CI: 0.34–0.37). No significant association was observed with anemia after adjustment.

**Conclusion:**

Delayed motherhood among women aged 40–49 in India is associated with maternal vulnerabilities, higher underweight prevalence, unmet contraceptive needs, and elevated pregnancy termination rates, alongside lower sterilization and overweight prevalence. These findings underscore the need for age-responsive and culturally sensitive maternal and reproductive health policies ensuring comprehensive prenatal, postnatal care and robust family planning services, empowering women to make informed reproductive choices in context of advanced maternal age.

## Introduction

Maternal age at childbirth is a key demographic and public health indicator that reflects broader socio-economic processes and shapes women's health outcomes throughout the life course. Over recent decades, many regions of the world have experienced a steady increase in the age at first birth, driven by shifts in educational attainment, labor force participation, marriage patterns, and expanded access to reproductive health services (1, 2). These trends have contributed to the rise of “delayed motherhood,” commonly operationalized as first childbirth at age 35 years or older, a phenomenon that has gained increasing research and policy attention (3, 4).

While much of the literature on delayed motherhood originates from high-income settings, where advanced maternal age has been linked to clinical outcomes and fertility intentions (3, 4), there is growing recognition that socio-economic and health-system factors significantly shape reproductive timing and outcomes in low- and middle-income countries (LMICs) as well (5, 6). Recent studies in diverse LMIC contexts indicate that delayed childbirth is influenced by cumulative socio-economic disadvantage, educational gradients, and uneven access to family planning and maternal healthcare services (5, 6). These findings suggest that conceptualizations rooted solely in biomedical risk frameworks may not adequately capture the lived realities of women in resource-constrained settings.

In India, delays in childbearing are emerging alongside broader demographic and social transitions. Traditionally characterized by early marriage and early childbearing, India has seen gradual increases in the age at marriage and first birth over the past two decades (7). According to the National Family Health Survey (NFHS-5, 2019–21), the median age at first birth increased from 19.8 years in NFHS-3 (2005–06) to 21.2 years in NFHS-5 (2019–21), with a notable rise in the proportion of women initiating motherhood after age 30 (7). Recent analyses further document that age at last birth and the overall reproductive age profile of Indian women are shifting upward, particularly among urban, educated, and economically better-off women (8–11). Singh et al. (8, 9) specifically highlight changes in age at last birth and broader shifts in delayed motherhood across India, emphasizing socio-economic determinants and cohort-period effects (10, 11). These patterns reflect changing fertility norms, increasing educational attainment, and expanded participation of women in higher education and the workforce (12, 13).

Expanding access to modern contraceptive methods and reproductive health services has also enabled greater control over the timing of pregnancies, contributing to delayed childbearing in some subpopulations (14, 15). However, substantial socio-economic and geographic disparities persist in access to high-quality family planning and maternal healthcare in India, with rural, low-income, and marginalized groups facing persistent barriers to services (16, 17). These inequalities underscore the importance of analyzing delayed motherhood not merely as a fertility timing choice but as a phenomenon deeply embedded in structural conditions and health system performance.

In contrast to a biomedical focus on obstetric risk, evidence increasingly underscores nutritional status, long-term socio-economic conditions, and healthcare access as critical determinants of maternal health outcomes across reproductive ages. For example, studies using NFHS data show persistent undernutrition and high anemia prevalence among reproductive-age women in India, often correlated with wealth disparities, education levels, and rural residence (18, 19). Other research highlights complex interactions between fertility behavior, contraceptive use, and reproductive health service engagement, pointing to the multifaceted nature of reproductive trajectories in low-resource settings (20, 21).

Despite these trends, there remains a paucity of research that directly examines the associations between delayed motherhood and maternal health indicators such as nutritional status, contraceptive behavior, pregnancy termination, and permanent contraception uptake in India (22, 23). Some study provided evidence on broader shifts in delayed motherhood in India using age-period-cohort and join point analyses, underscoring the need to consider cohort and period effects in studying maternal health outcomes (10, 11).

The present study aims to investigate the association between delayed motherhood and selected maternal health outcomes in India, with an emphasis on maternal nutritional status and reproductive health service utilization. By shifting analytical focus from solely biomedical risk to include socio-economic and healthcare-access dimensions, this study seeks to provide context-specific evidence to inform public health policy and reproductive health programming tailored to the needs of women in India.

## Data and methodology

### Data source

We utilized data from the fifth National Family Health Survey (NFHS-5), a comprehensive and nationally representative household survey conducted across all states and union territories in India from 2019 to 2021 (24). The primary objective of the survey was to gather accurate and up-to-date information on various health and demographic indicators, including maternal and child health, fertility, childhood mortality and morbidity, family planning practices, immunization, nutritional status, pregnancy-related issues, healthcare utilization, non-communicable diseases, domestic violence, and breastfeeding practices (24, 25) Additionally, NFHS-5 provided valuable data regarding the ages at which various reproductive events occurred, such as age at first marriage, age at first cohabitation, age at first sexual intercourse, age at first birth, age at most recent birth, and more. The survey was conducted in two phases. Phase-I took place from 17 June 2019 to 30 January 2020, covering 17 states and 5 Union Territories. Phase II was conducted from 2 January 2020 to 30 April 2021, covering 11 states and 3 Union Territories. Thus, NFHS-5 provides information for 707 districts, 28 states, and 8 union territories. The NFHS-5 survey sample was designed as a stratified two-stage sample. The 2011 census data served as the sampling frame for the selection of Primary Sampling Units (PSUs). In rural areas, the PSUs were villages, and in urban areas, the PSUs were Census Enumeration Blocks (CEBs). A total of 17 Field Agencies gathered information from 636,699 households, 724,115 women, 101,839 men, and 232,920 births that occurred during the last 5 years leading up to the survey. NFHS-5 has proven to be a crucial source of data, offering insights into various aspects of public health and demographics in India, aiding policymakers and researchers in making informed decisions, and addressing critical issues related to health and well-being (24, 25).

### Sample selection

**Figure 1:**
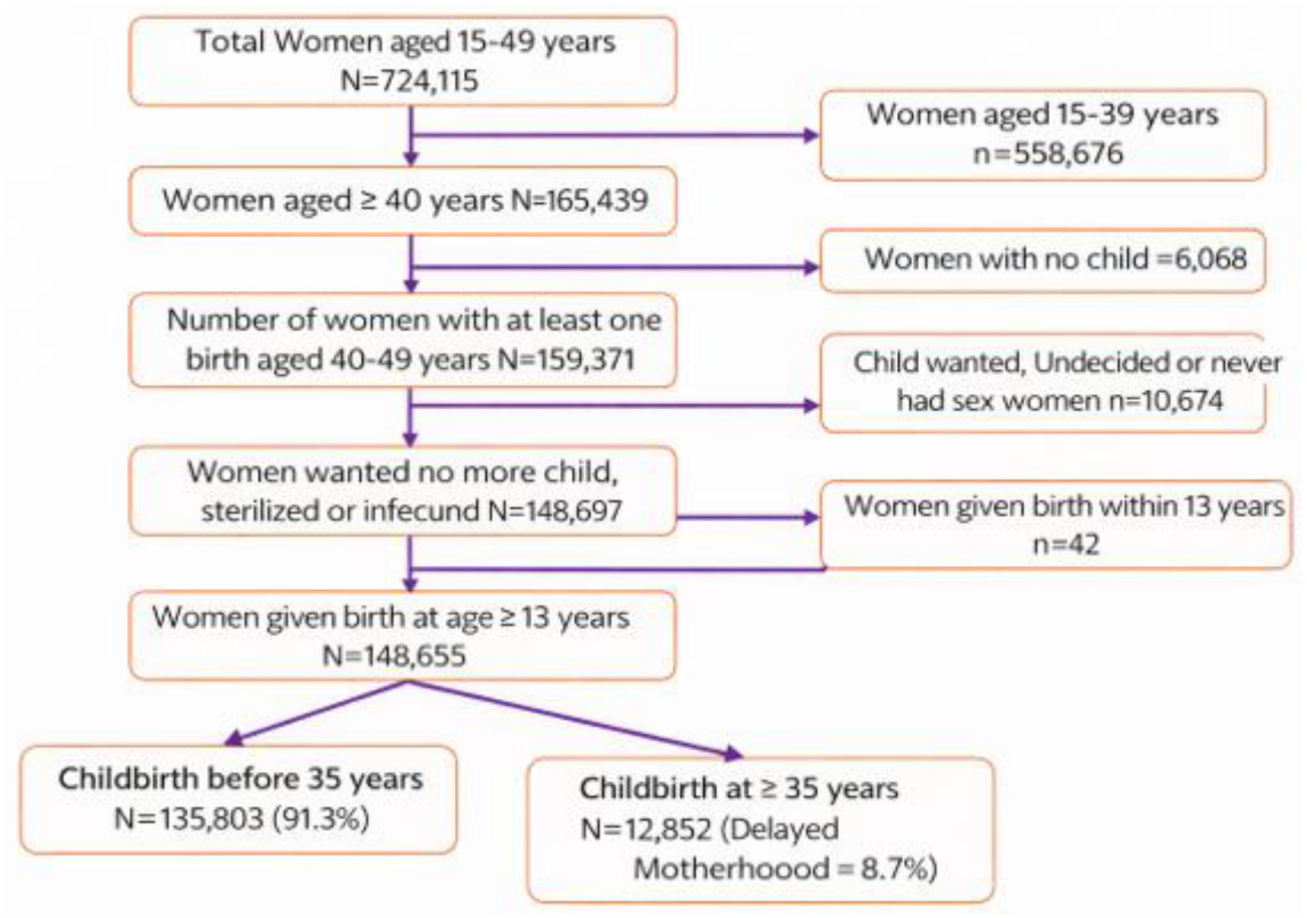
Flow diagram depicting the sample selection process for delayed motherhood in India 2019–21.

The study sample was drawn from the National Family Health Survey-5 (NFHS-5), 2019–21, which interviewed 724,115 women aged 15–49 years across India. For the present analysis, women aged 15–39 years (*n* = 558,676) were excluded to focus on nearly complete reproductive experience so that her most recent birth would be the last birth. Among women aged 40–49 years (*n* = 165,439), those with no children (*n* = 6,068) were excluded. The analysis was further restricted to women who had at least one live birth (*n* = 159,371). Women who reported wanting more children, were undecided, or had never had sexual intercourse (*n* = 10,674) were excluded to ensure that the most recent birth reflected a completed or near-completed fertility experience. Additionally, women who reported a birth before age 13 (*n* = 42) were excluded due to data quality concerns. The final analytical sample comprised 148,655 women aged 40–49 years with at least one live birth. Delayed motherhood was defined based on the age at the most recent childbirth which is probably the last birth: women whose last birth occurred at age ≥35 years were classified as experiencing delayed motherhood (*n* = 12,852; 8.7%), while those whose last birth occurred before age 35 constituted the reference group (*n* = 135,803; 91.3%) (Figure 1). Importantly, this classification was independent of parity; the most recent birth could be of any birth order, and delayed motherhood was defined solely based on maternal age at the time of the latest childbirth rather than whether it was a first or higher-order birth. Furthermore, women aged 40–49 years were selected to represent a completed fertility cohort, ensuring that the most recent childbirth is likely to reflect the last birth (22, 23).

## Variable description

### Outcome variables

Various maternal health indicators are the outcomes of interest in this study. Maternal health indicators for nutritional status encompass underweight, overweight, and anemia status (Any Anemia, Mild Anemia, Moderate Anemia, Severe Anemia). Health care utilization indicators for women consist the unmet need. As well as indicators related to pregnancy and contraception are ever-terminated pregnancy, contraceptive pill use before the first childbirth, current modern contraception use, and sterilization. By analyzing these various indicators, the study aims to gain insights into the health status and healthcare utilization of women, focusing on nutrition, unmet need, and pregnancy-related outcomes.

### Predictor variables

During the survey, respondents were asked to provide month and year of birth for each of their children. This information was used to calculate the exact maternal age at each childbirth, including the most recent birth. Maternal age at last (most recent) childbirth was derived by subtracting the respondent's date of birth from the reported month and year of the child's birth. The primary predictor variable, delayed motherhood was constructed as a dichotomous variable and defined as having the most recent childbirth at age ≥35 years (“yes”) or < 35 years (“no”), irrespective of parity. Using this classification, the study examined the association between delayed motherhood and selected maternal health indicators.

### Control variables

The study included several socioeconomic and demographic variables as control variables for estimating the effect of delayed motherhood on maternal health indicators. While estimating the effect on maternal health indicators, the control variables considered were: place of residence, religion, caste, age of the respondent, education, wealth index, exposure to mass media, toilet facility, and drinking water facility. By including these control variables, the study aimed to account for potential confounding factors and ensure that the effects of delayed motherhood and other relevant factors on maternal health indicators could be more accurately assessed.

### Statistical analysis

To investigate the associations between delayed motherhood and maternal outcomes, bivariate analysis and a generalized linear model were employed. The model assumed a binomial distribution and used a Logit link for the binary outcome. The initial assessment of associations was done using chi-square tests. Subsequently, a generalized linear model with a binomial distribution assumption and a Logit link for the binary outcome was utilized to determine the risk of maternal health indicators in response to the timing of last birth. Before fitting the model, the potential multi-collinearity between independent variables was evaluated using the variance inflation factor (VIF) to ensure the accuracy and reliability of the results. To account for the complex survey design of NFHS-5, including stratification and clustering, the data were set using Stata's svyset command with primary sampling units (PSUs), sampling strata, and normalized probability weights (v005/1,000,000). All generalized linear models were estimated using the svy: glm command, ensuring that standard errors, confidence intervals, and *P*-values were calculated using Taylor series linearization. Degrees of freedom for variance estimation were based on the number of PSUs minus the number of strata, yielding nationally representative and statistically valid estimates. The model's output presented the adjusted odds ratio of maternal health indicators among selected reproductive-aged women, along with their corresponding 95 percent confidence intervals (95 percent CI). A significance level of *P* < 0.05 was considered statistically significant. For conducting the analyses, the data were analyzed using Stata software, specifically version 16.0, developed by Stata Corp LLC, based in College Station, TX. (26, 27).

## Results

**Figure 2:**
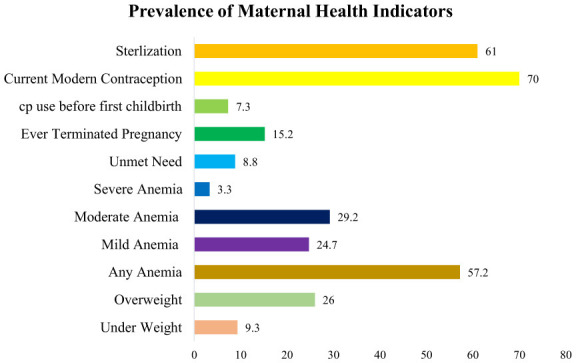
Prevalence of maternal health indicators among women aged 40–49 in India.

Figure 2 illustrates the prevalence of maternal health indicators related to nutrition status, healthcare utilization, and contraception among women aged 40–49 years. The results reveal that approximately 9 percent of these women were underweight, while a significant portion, about 26 percent, were overweight. Additionally, more than half of the women, specifically 57.2 percent, were found to have some form of anemia, with 24.7 percent experiencing mild anemia and 29.2 percent exhibiting moderate anemia. Figure 2 also highlights that only 7.3 percent of women utilized contraception before their first childbirth, while approximately 70 percent of women are currently using modern contraception methods. These findings shed light on the diverse maternal health challenges and practices within this age group.

The sample characteristics of chosen women between the ages of 40 and 49 are shown in Table 1, which also shows an association between women health outcomes and control variables with delayed motherhood. In our sample, the prevalence of delayed motherhood was 8.7% (as shown in Table 1). Furthermore, the prevalence of underweight (14.0%), any form of anemia (57.9%), mild anemia (24.9%), and moderate anemia (29.9%) was notably higher among women who experienced delayed motherhood. Despite the elevated rates of anemia among delayed motherhood women, no significant associations were observed in any of these cases. Similarly, unmet need (15.9%), previous pregnancy termination (19.2%), and contraception use before their first childbirth (9.1%) were more common among delayed motherhood women. All indicators related to pregnancy and contraception were significantly associated with delayed motherhood. Likewise, all other control variables displayed significant associations with delayed motherhood.

**Table 1:** Sample characteristics of selected women aged 40–49 years.

**Background variables**	**Delayed motherhood**			
	**Total**	**No**	**Yes**	***P*** **-value**
**Outcome variables**				
**Nutritional status**				
Under weight	13,417 (9.3)	11,682 (8.9)	1,735 (14.0)	< 0.001
Over weight	37,417 (26)	34,825 (26.4)	2,593 (20.9)	< 0.001
Any anemia	80,734 (57.2)	73,647 (57.1)	7,088 (57.9)	0.051
Mild anemia	34,913 (24.7)	31,859 (24.7)	3,054 (24.9)	0.979
Moderate anemia	41,193 (29.2)	37,534 (29.1)	3,659 (29.9)	0.022
Severe anemia	4,628 (3.3)	4,254 (3.3)	375 (3.1)	0.768
**Access to health care utilization**				
Unmet need	13,063 (8.8)	11,019 (8.1)	2,044 (15.9)	< 0.001
**Pregnancy and contraception related**				
Ever terminated pregnancy	22,516 (15.2)	20,054 (14.8)	2,462 (19.2)	< 0.001
Contraceptive pills use before first childbirth	9,509 (7.3)	8,548 (7.1)	961 (9.1)	< 0.001
Current modern contraception	103,999 (70)	97,568 (71.9)	6,431 (50.0)	< 0.001
Sterilization	90,705 (61)	86,220 (63.5)	4,485 (34.9)	< 0.001
**Control variables**				
**Residence**				
Urban	51,133 (34.4)	47,613 (35.1)	3,520 (27.4)	< 0.001
Rural	97,522 (65.6)	88,190 (64.9)	9,332 (72.6)	
**Caste**				
Sc	31,139 (22.0)	28,293 (21.8)	2,846 (23.3)	< 0.001
St	12,919 (9.1)	11,389 (8.8)	1,530 (12.5)	
Obc	63,958 (45.1)	58,622 (45.2)	5,337 (43.7)	
Others	33,876 (23.9)	31,365 (24.2)	2,511 (20.5)	
**Religion**				
Hindu	123,425 (83.1)	113,889 (83.9)	9,536 (74.2)	< 0.001
Muslims	16,831 (11.3)	14,250 (10.5)	2,580 (20.1)	
Christian	3,880 (2.6)	3,443 (2.5)	437 (3.4)	
Others	4,433 (3.0)	4,142 (3.1)	291 (2.3)	
**Respondent current age**				
40–44	72,713 (48.9)	67,667 (49.8)	5,046 (39.3)	< 0.001
45–49	75,943 (51.1)	68,137 (50.2)	7,806 (60.7)	
**Education**				
No education	65,709 (44.2)	58,195 (42.9)	7,514 (58.5)	< 0.001
Primary	23,584 (15.9)	22,118 (16.3)	1,466 (11.4)	
Secondary	48,785 (32.8)	46,120 (34.0)	2,665 (20.7)	
Higher	10,577 (7.1)	9,370 (6.9)	1,207 (9.4)	
**Wealth index**				
Poorest	24,749 (16.7)	20,098 (14.8)	4,651 (36.2)	< 0.001
Poorer	28,033 (18.9)	25,358 (18.7)	2,676 (20.8)	
Middle	30,845 (20.8)	28,931 (21.3)	1,915 (14.9)	
Richer	31,446 (21.2)	29,798 (21.9)	1,648 (12.8)	
Richest	33,581 (22.6)	31,619 (23.3)	1,962 (15.3)	
**Exposure to mass media**				
No	37,946 (25.5)	32,317 (23.8)	5,630 (43.8)	< 0.001
Any	110,709 (74.5)	103,487 (76.2)	7,222 (56.2)	
**Toilet facility**				
Unimproved	28,198 (19.1)	24,350 (18.1)	3,849 (30.1)	< 0.001
Improved toilet	119,396 (80.9)	110,467 (81.9)	8,929 (69.9)	
**Drinking water facility**				
Unimproved	10,352 (7.0)	9,634 (7.2)	718 (5.6)	< 0.001
Improved water	137,242 (93.0)	125,183 (92.8)	12,059 (94.4)	
**Regions**				
East	31,738 (21.4)	28,336 (20.9)	3,403 (26.5)	< 0.001
West	22,397 (15.1)	21,252 (15.7)	1,145 (8.9)	
North	20,385 (13.7)	18,893 (13.9)	1,493 (11.6)	
South	35,948 (24.2)	34,527 (25.4)	1,420 (11.1)	
Central	33,051 (22.2)	28,528 (21.0)	4,523 (35.2)	
Northeast	5,136 (3.5)	4,268 (3.1)	868 (6.8)	
Total	148,655	135,803 (91.3)	12,852 (8.7)	

Total N may or may not be equal due to missing cases.

Table 2 presents estimate from the generalized linear model depicting the association between maternal health indicators and delayed motherhood in India. The table reveals significant associations between maternal health indicators and the delayed age at the last birth of the mother. The adjusted analysis shows that the risk of women being underweight (AOR: 1.15, 95% CI: 1.09, 1.22) is 15% higher among women who gave birth after the age of 35, while the likelihood of being overweight (AOR: 0.90, 95% CI: 0.86, 0.94) is 10% lower in this group. Furthermore, adjusted estimates indicate that the probability of experiencing unmet need (AOR: 1.98, 95% CI: 1.89–2.09), pregnancy termination (AOR: 1.43, 95% CI: 1.36–1.50), and contraception use before the first childbirth (AOR: 1.23, 95% CI: 1.15–1.32) is significantly higher among women who had their last birth after the age of 35, with odds ratios of 1.98, 1.43, and 1.23, respectively. Similarly, women in this age group were 65% less likely to undergo sterilization (AOR: 0.35, 95% CI: 0.34–0.37). All adjusted estimates were controlled for residence, caste, religion, respondent age, education, wealth index, exposure to mass media, toilet facility, and drinking water facility.

**Table 2:** Generalized linear model estimates showing the relationship between maternal health indicators and delayed age at Last Birth in India, 2019–21.

**Indicators of health**	**COR (95% CI)**	**AOR (95% CI)**
**Maternal health indicators** ^+^		
**Nutritional status**		
Under weight	1.51^***^ [1.44, 1.59]	1.15^***^ [1.09, 1.22]
Overweight	0.75^***^ [0.72, 0.78]	0.90^***^ [0.86, 0.94]
Any anemia	1.03 [1.00, 1.07]	1.01 [0.97, 1.05]
Mild anemia	0.99 [0.96, 1.04]	0.99 [0.95, 1.03]
Moderate anemia	1.05^*^ [1.01, 1.08]	1.02 [0.98, 1.06]
Severe anemia	0.99 [0.89, 1.09]	0.99 [0.89, 1.10]
**Access to health care utilization**		
Unmet need	2.06^***^ [1.97, 2.16]	1.98^***^ [1.89, 2.09]
**Pregnancy and contraception related**		
Ever terminated pregnancy	1.30^***^ [1.25, 1.36]	1.43^***^ [1.36, 1.50]
Contraceptive pills use before first childbirth	1.25^***^ [1.18, 1.34]	1.23^**^ [1.15, 1.32]
Current modern contraception	0.42^***^ [0.40, 0.43]	0.49^***^ [0.47, 0.51]
Sterilization	0.32^***^ [0.31, 0.33]	0.35^***^ [0.34, 0.37]

^+^indicates that maternal health indicators for Adjusted analyses were controlled for residence, caste, religion, respondent age, education, wealth index, exposure to mass media, toilet facility, and drinking water facility.

COR, Crude odds ratio; AOR, Adjusted odds ratio.

^**^p < 0.01; ^***^p < 0.001.

## Discussion

The present study examines the association between delayed motherhood (defined as childbirth at age 35 years or above) and selected maternal health and reproductive outcomes in India using a generalized linear model. The findings indicate that delayed motherhood is significantly associated with a higher likelihood of being underweight, increased use of contraception prior to first childbirth, a greater probability of pregnancy termination, and a substantially lower likelihood of undergoing sterilization. Importantly, these associations predominantly reflect socio-behavioral patterns and healthcare access dynamics, highlighting the need to situate delayed motherhood in India within broader life-course, socio-economic, and health-system contexts rather than relying on interpretations derived from high-income countries (10, 11).

One of the most striking findings of this study is the higher likelihood of underweight among women who experienced delayed motherhood. This result is counterintuitive when compared with evidence from Western contexts, where delayed childbearing is often associated with overweight or obesity due to sedentary lifestyles, dietary patterns, and cumulative exposure to obesogenic environments (28, 29). In contrast, in India, delayed motherhood may reflect a very different socio-economic profile. Recent demographic research suggests that women who give birth at later ages are not necessarily socio-economically privileged, but may instead experience delayed marriage, spousal absence, marital disruption, or prolonged economic insecurity (30–33). These factors are closely associated with chronic nutritional deprivation and limited access to preventive healthcare, which can manifest as underweight status in later reproductive ages (33, 34).

Furthermore, the observed association between delayed motherhood and underweight may be influenced by parity-related confounding and cumulative reproductive stress across the life course. Many women who give birth at older ages may have already experienced multiple pregnancies earlier in life, often under conditions of inadequate nutrition and short birth intervals. Repeated pregnancies without sufficient nutritional replenishment are known to deplete maternal energy reserves, particularly in low-resource settings, increasing the risk of undernutrition later in life (15, 35). Studies from India and other low- and middle-income countries have consistently shown that high parity and closely spaced births are strongly associated with poor maternal nutritional outcomes, especially among socio-economically disadvantaged women (36, 37). Thus, underweight among women with delayed motherhood should be interpreted as a marker of long-term structural disadvantage and accumulated nutritional stress, rather than as a direct consequence of maternal age (37).

The high prevalence of anemia observed among women with delayed motherhood further underscores the role of structural determinants of health. Although more than half of the women in this group were anemic, delayed motherhood was not independently associated with anemia after adjustment (15, 23). This finding aligns with national evidence demonstrating that anemia among Indian women is a pervasive public health problem, driven by chronic dietary inadequacy, micronutrient deficiencies, infections, and gender-based nutritional inequities, rather than reproductive timing alone (15, 23). Recent analyses using NFHS-5 data have shown that socio-economic status, education, and region explain substantially more variation in anemia prevalence than maternal age or parity (18, 19). These findings reinforce the interpretation that anemia reflects broader social and nutritional vulnerabilities rather than the timing of childbirth (18, 19).

The study also reveals important patterns in reproductive health service utilization among women who delayed motherhood. Women in this group were more likely to have used contraception prior to their first childbirth, suggesting some engagement with family planning services earlier in the reproductive life course (14, 34). However, the simultaneous increase in unmet need for contraception and pregnancy termination points to inconsistent, discontinuous, or ineffective access to contraceptive methods (14, 15). This pattern reflects longstanding challenges within India's family planning program, including method-specific biases, limited counseling, and inadequate follow-up, particularly for older and socio-economically disadvantaged women (14, 15, 34). Pregnancy termination in this context may therefore indicate reliance on reactive reproductive health strategies when preventive contraceptive options are unavailable, unacceptable, or poorly supported (35, 36).

A particularly notable finding is the substantially lower likelihood of sterilization among women who delayed motherhood. Female sterilization remains the dominant contraceptive method in India and is closely tied to early completion of childbearing and parity-based programmatic targeting (23). Women who delay childbirth may fall outside these conventional service delivery pathways, resulting in lower exposure to or uptake of permanent methods. While this pattern may partly reflect a preference to retain reproductive flexibility, it also highlights programmatic rigidity and gaps in age-inclusive family planning services that fail to accommodate diverse reproductive trajectories (36, 37). Recent studies emphasize that India's family planning system remains insufficiently responsive to women whose fertility pathways diverge from early marriage and early childbearing norms (11, 37).

Emerging demographic evidence further supports the interpretation that delayed motherhood in India is shaped by cohort effects, socio-economic inequality, and changing family formation patterns rather than uniform fertility postponement (10, 11). Singh et al. (8, 9) emphasize that delayed motherhood represents a heterogeneous phenomenon, encompassing women with constrained reproductive choices as well as those navigating evolving fertility preferences (10, 11). Recognizing this heterogeneity is essential for interpreting maternal health outcomes and designing responsive public health interventions (10, 11, 33, 38).

Overall, delayed childbearing among women aged 40–49 years in India reflects cumulative socio-economic disadvantage, long-term nutritional vulnerability, and fragmented engagement with reproductive health services across the life course. The associations observed in this study highlight important gaps in the inclusiveness, continuity, and equity of nutrition and family planning services for women who do not follow early childbearing trajectories. Addressing these challenges requires public health strategies that strengthen nutrition support, expand access to a broad range of modern contraceptive methods, and ensure age-inclusive reproductive healthcare that accommodates diverse reproductive pathways. Future research should continue to explore these interconnected social and health-system determinants to inform policies aimed at reducing structural inequities and improving maternal health outcomes among women who delay motherhood in India (10, 11, 14, 15, 15, 18, 19, 23, 28–34, 34, 35, 35, 36, 36, 37, 37).

## Conclusion

The study highlights the heightened risks associated with delayed motherhood, emphasizing the need for specialized care for women who choose to conceive later in life. By incorporating control variables such as place of residence, religion, caste, age, education, wealth index, and access to media and sanitation facilities, the analysis accounted for potential confounding factors that could influence maternal health outcomes. Although delayed motherhood occurs less frequently, pregnancies at an advanced maternal age are classified as high-risk, necessitating extra vigilance and care from healthcare providers. The findings stress the importance of developing targeted healthcare interventions that cater specifically to the needs of older first-time mothers. These interventions should include comprehensive prenatal and postnatal care to reduce the risks associated with late pregnancies. Additionally, there is a critical need to improve access to and education about reproductive health services across all life stages to empower women to make well-informed reproductive choices. In response to these needs, the introduction of pre-pregnancy care clinics is recommended. These clinics would provide pre-conception services to prospective mothers and their partners starting 3 months prior to conception. The services offered would focus on assessing and mitigating pregnancy risks, enhancing maternal health before pregnancy, and promoting healthy lifestyle choices for both partners. This proactive approach aims to improve overall maternal and fetal outcomes by ensuring that women entering pregnancy are as healthy as possible.

### Strength and limitations

This study draws on extensive literature to contextualize its findings, demonstrating a solid understanding of the research landscape. A large sample size provides sufficient power to detect significant associations, and inclusion of demographic and socioeconomic control variables reduces potential confounding. The use of a generalized linear model allows for advanced statistical analysis, identifying associations between delayed motherhood and maternal health indicators while adjusting for multiple covariates.

However, several limitations should be noted. The cross-sectional design based on retrospective birth history data limits causal inference and recall bias may affect variables such as age at childbirth and timing of contraceptive use, particularly among older women. Potential conflation between “first” and “last” births could also influence birth-order interpretations. The findings may not fully generalize beyond the study population due to variations in healthcare access, cultural norms, and socioeconomic contexts. Reliance on selected control variables may introduce selection bias if they do not fully represent the broader population. While associations are identified, a deeper exploration of specific health outcomes and their mechanisms would strengthen the analysis.

Overall, the study provides valuable insights into delayed motherhood, but results should be interpreted cautiously. Future prospective or longitudinal research, as well as qualitative studies, could offer stronger evidence on causality and underlying pathways, guiding targeted interventions and policies.

## Data Availability

Publicly available datasets were analyzed in this study. This data can be found here: The data is freely available in the public domain and survey agencies that conducted the field survey for the data collection have collected prior consent from the respondent. It also guaranteed that the participants' privacy was protected and that informed consent was obtained from respondents during the survey. Therefore, prior ethical approval for using the datasets was not required. The dataset is accessible to registered users at: https://www.dhsprogram.com/data/available-datasets.cfm.

## References

[1] Sobotka T Beaujouan É. Two-child families in Europe: evidence from the past three decades. Popul Stud. (2018) 72:21–40. doi: 10.1553/0x003d069

[2] Balasch J Gratacós E. Delayed childbearing: effects on fertility and the outcome of pregnancy. Fertil Steril. (2019) 111:223–31. doi: 10.1097/GCO.0b013e328351790821228557

[3] Myrskylä M van Raalte A Elo IT. The association between advanced maternal age and adverse pregnancy outcomes: evidence from high-income countries. Demography. (2013) 50:1183–206. doi: 10.1007/s13524-013-0217-4

[4] Schmidt L Sobotka T Bentzen JG Nyboe Andersen A. Demographic and medical consequences of the postponement of parenthood. Hum Reprod Update. (2012) 18:29–43. doi: 10.1093/humupd/dmr040 21989171

[5] Zhu W Zhang W Chen Y. Delayed motherhood in low- and middle-income countries: socio-economic determinants and policy implications. BMC Pregnancy Childbirth. (2018) 18:450.30458752

[6] Sugranyes G Houghton L Crowley P. Socio-economic factors affecting reproductive timing in developing countries. Reprod Health. (2020) 17:120.32787868

[7] International Institute for Population Sciences (IIPS), ICF. National Family Health Survey (NFHS-5), 2019–21: India. Mumbai: IIPS (2021).

[8] Singh A Sharma P Kumar V. Shifts in reproductive age profile and implications for maternal health in urban India. J Biosoc Sci. (2023) 55:615–32. doi: 10.1017/S0021932022000376

[9] Singh A Kumar R Ghosh S. Trends in age at first and last birth in India: socioeconomic differentials. Popul Rev. (2025) 64:101–23.

[10] Singh M Shekhar C Shri N. Changes in age at last birth and its determinants in India. Sci Rep. (2023) 13:10450. doi: 10.1038/s41598-023-37370-z 37369774 PMC10300096

[11] Singh M Shekhar C Gupta J Barik S. Exploring the shifting landscape of delayed motherhood in India: a comprehensive analysis using joinpoint and age-period-cohort analysis. BMC Womens Health. (2025) 25:563. doi: 10.1186/s12905-025-04104-4 41257671 PMC12628530

[12] Goli S Arokiasamy P Singh D. Education, work, and delayed childbearing in India. Demogr Res. (2018) 39:1125–48.

[13] Kaur R Prakash R. Women's labor force participation and delayed fertility in India. Asian Popul Stud. (2022) 18:34–50.

[14] Cleland J Conde-Agudelo A Peterson H Ross J Tsui A. Contraception and health. Lancet. (2018) 391:260–76. doi: 10.1016/S0140-6736(18)30009-7 22784533

[15] Sen B Kumar V Tripathi R. Family planning access and delayed motherhood in India: evidence from NFHS. Indian J Public Health. (2024) 68:12–20.

[16] Kumar S Singh A Dey S. Socio-economic disparities in access to reproductive health services in India. Health Policy Plan. (2019) 34:531–40. doi: 10.1093/heapol/czz059

[17] Dey S Sharma M Kaur S. Rural-urban inequities in maternal health service utilization: evidence from India. BMC Health Serv Res. (2021) 21:112. doi: 10.1186/s12913-021-06122-533530994

[18] Menon P Kalita D Bhatia K. Nutritional status of reproductive-age women in India: trends from NFHS. Public Health Nutr. (2023) 26:615–27.

[19] Bentley ME Griffiths PL. Underweight and anemia among Indian women: socio-economic and geographic disparities. Matern Child Nutr. (2021) 17:e13145.33528101

[20] Singh D Sharma P Ghosh S. Fertility behavior, contraceptive use, and reproductive health engagement in India. Reprod Health. (2022) 19:143.35725562

[21] Mishra R. Reproductive trajectories and healthcare access in low-resource settings. J Fam Reprod Health. (2025) 19:23–36.

[22] Patra S. Delayed motherhood in India: trends and implications. Indian J Demography. (2019) 54:101–15.

[23] Chaurasia AR. Reproductive health patterns and delayed childbirth in India. Popul Stud. (2020) 74:345–60.

[24] International Institute for Population Sciences (IIPS) & ICF. NFHS-5 Data File Documentation. Mumbai: IIPS; 2021.

[25] International Institute for Population Sciences (IIPS). Sampling design and methodology of NFHS-5. Mumbai: IIPS (2021).

[26] StataCorp. Stata Statistical Software: Release 16. College Station, TX: StataCorp LLC (2019).

[27] Sarwer DB Allison KC Martino C Wadden TA. Delayed pregnancy and maternal BMI: evidence from Western countries. Obesity Rev. (2006) 7:235–43.

[28] Alemu S Umeta M. Maternal nutritional stress and delayed childbearing in LMICs. BMC Pregnancy Childbirth. (2015) 15:303.26589617

[29] Rahman A Rahman M Hossain M. Cumulative reproductive stress and maternal underweight in South Asia. Matern Child Health J. (2020) 24:1345–55.32876813

[30] Bongaarts J. High parity, socio-economic disadvantage, and maternal health outcomes. Popul Stud. (2015) 69:143–58.

[31] Black RE Victora CG Walker SP Bhutta ZA Christian P de Onis M . Maternal and child undernutrition and overweight in low-income and middle-income countries. Lancet. (2013) 382:427–51. doi: 10.1016/S0140-6736(13)60937-X 23746772

[32] International Institute for Population Sciences (IIPS), ICF. NFHS-5 Key Findings Report. Mumbai: IIPS (2021).

[33] Cleland J Ndugwa R Zulu E. Family planning dynamics and delayed motherhood in India. Popul Stud. (2012) 66:243–58.

[34] Moeller J Vandemoortele T Shah A. Reproductive health services and delayed fertility. Int Perspect Sex Reprod Health. (2019) 45:105–13.

[35] Leonard K. Pregnancy termination patterns in India. Stud Fam Plann. (1984) 15:115–24.

[36] Menken J. Maternal age and reproductive outcomes in low-resource settings. Popul Dev Rev. (1985) 11:87–104.

[37] Harris L Singh M Shekhar C. Age-inclusive family planning services in India. Reprod Health. (2021) 18:110.34078408

[38] Linne Y Dye L Barkeling B Rossner S. Lifestyle, weight, and delayed childbearing in Western populations. Int J Obes Relat Metab Disord. (2003) 27:456–62. doi: 10.1038/sj.ijo.0802441 14634683

